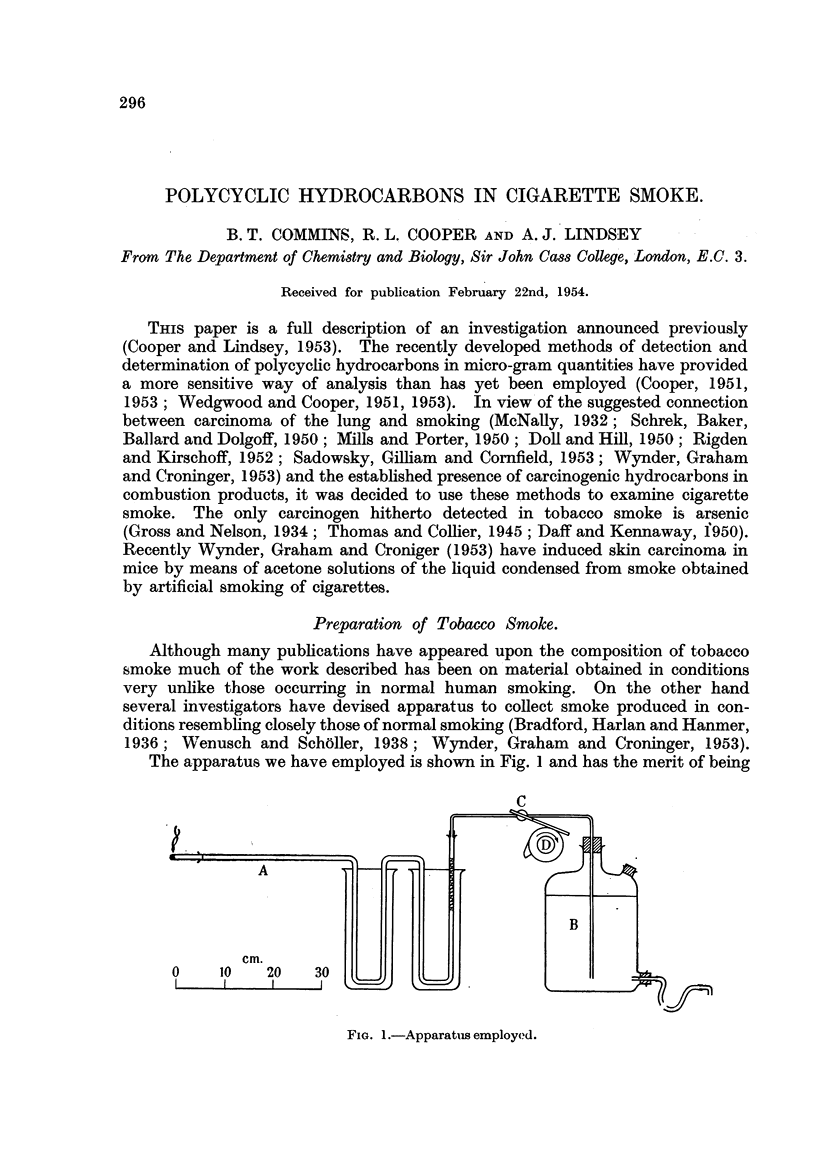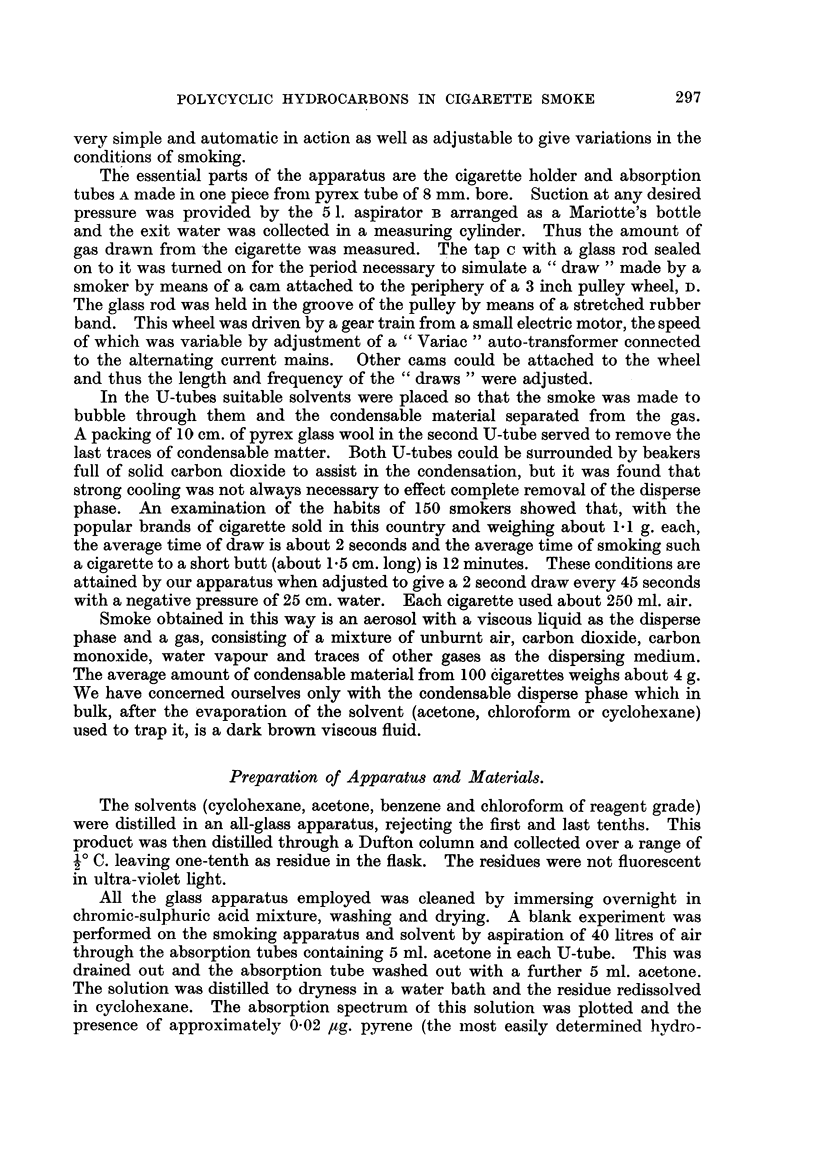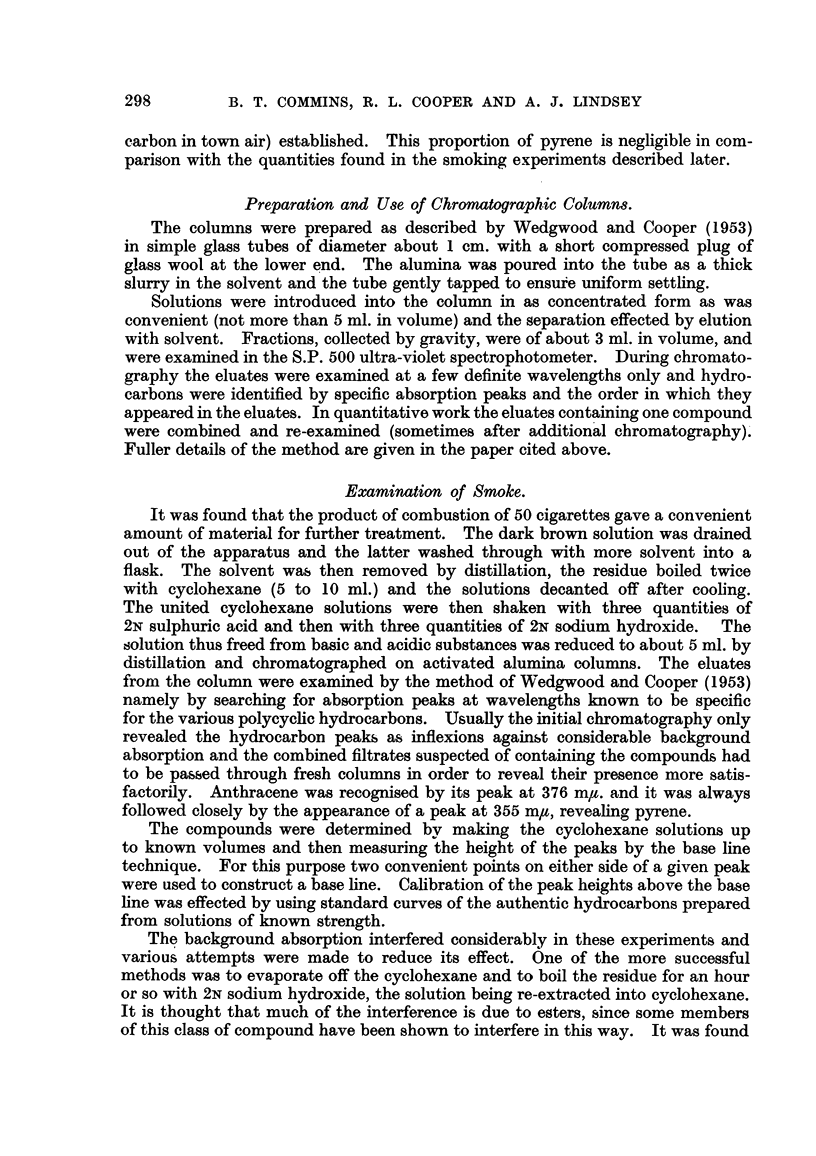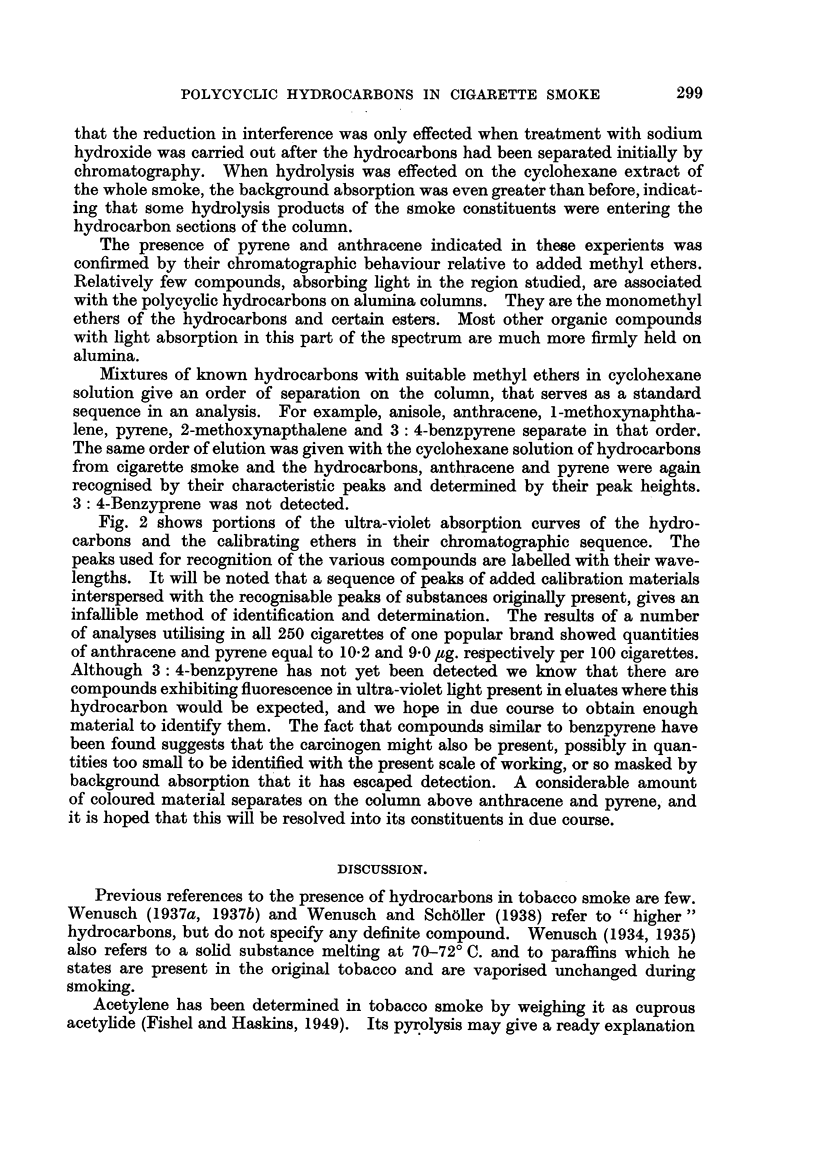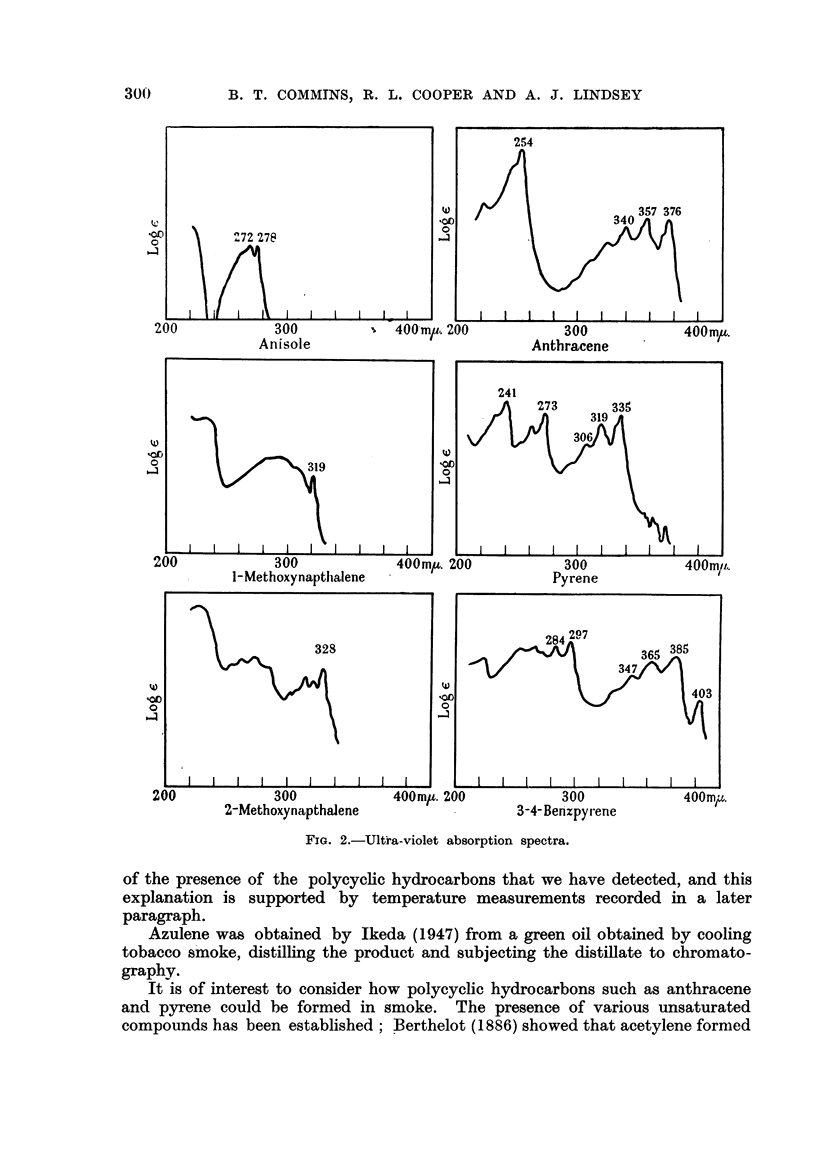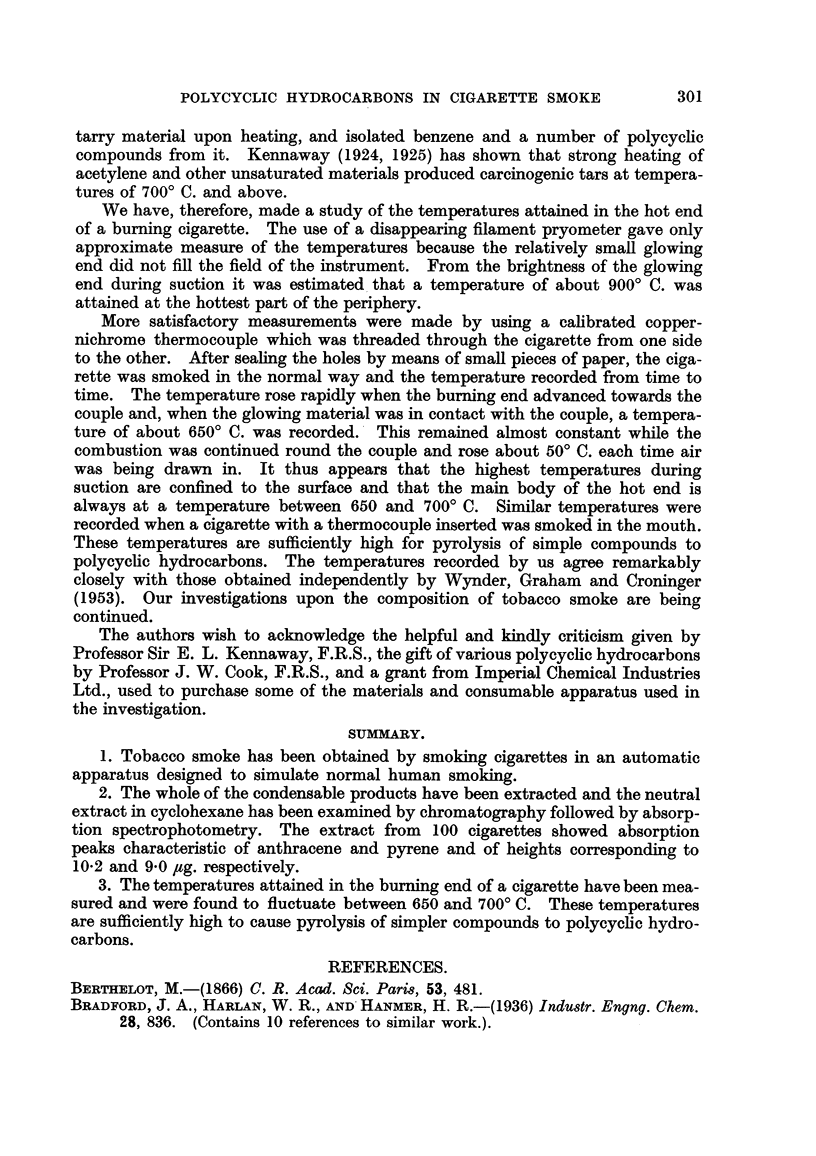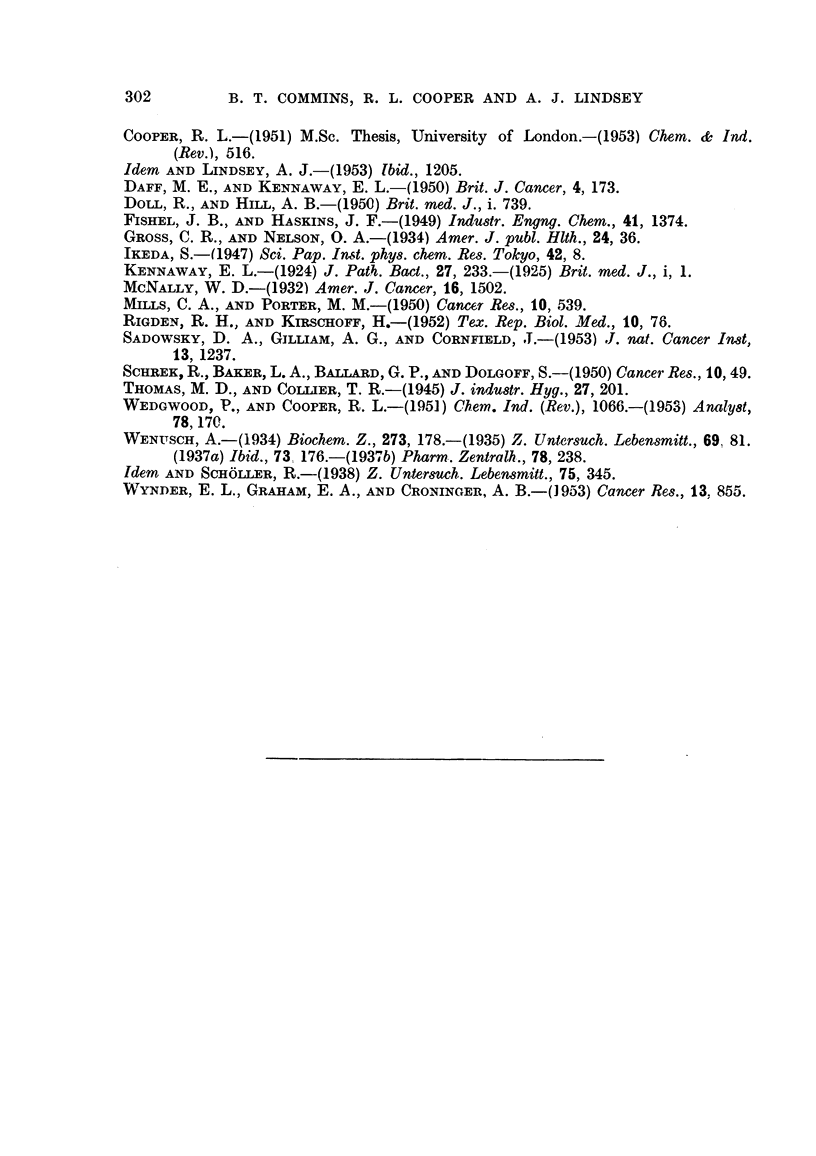# Polycyclic Hydrocarbons in Cigarette Smoke

**DOI:** 10.1038/bjc.1954.30

**Published:** 1954-06

**Authors:** B. T. Commins, R. L. Cooper, A. J. Lindsey


### POLYCYCLIC HYDROCARBONS IN CIGARETTE SMOKE.

B. T. COMMINS, R. L. COOPER AND A. J. LINDSEY
*From The Department of Chemistry and Biology, Sir John Cass College, London, E.C. 3.*

Received for publication February 22nd, 1954.

THIS paper is a full description of an investigation announced previously (Cooper and Lindsey, 1953). The recently developed methods of detection and determination of polycyclic hydrocarbons in micro-gram quantities have provided a more sensitive way of analysis than has yet been employed (Cooper, 1951, 1953 ; Wedgwood and Cooper, 1951, 1953). In view of the suggested connection between carcinoma of the lung and smoking (McNally, 1932 ; Schrek, Baker, Ballard and Dolgoff, 1950 ; Mills and Porter, 1950 ; Doll and Hill, 1950 ; Rigden and Kirschoff, 1952 ; Sadowsky, Gilliam and Cornfield, 1953 ; Wynder, Graham and Croninger, 1953) and the established presence of carcinogenic hydrocarbons in combustion products, it was decided to use these methods to examine cigarette smoke. The only carcinogen hitherto detected in tobacco smoke is arsenic (Gross and Nelson, 1934 ; Thomas and Collier, 1945 ; Daff and Kennaway, 1950). Recently Wynder, Graham and Croniger (1953) have induced skin carcinoma in mice by means of acetone solutions of the liquid condensed from smoke obtained by artificial smoking of cigarettes.

### *Preparation of Tobacco Smoke.*

Although many publications have appeared upon the composition of tobacco smoke much of the work described has been on material obtained in conditions very unlike those occurring in normal human smoking. On the other hand several investigators have devised apparatus to collect smoke produced in conditions resembling closely those of normal smoking (Bradford, Harlan and Hanmer, 1936 ; Wenusch and Schöller, 1938 ; Wynder, Graham and Croninger, 1953). The apparatus we have employed is shown in Fig. 1 and has the merit of being

FIG. 1.—Apparatus employed.

very simple and automatic in action as well as adjustable to give variations in the conditions of smoking.

The essential parts of the apparatus are the cigarette holder and absorption tubes A made in one piece from pyrex tube of 8 mm. bore. Suction at any desired pressure was provided by the 5 l. aspirator B arranged as a Mariotte's bottle and the exit water was collected in a measuring cylinder. Thus the amount of gas drawn from the cigarette was measured. The tap C with a glass rod sealed on to it was turned on for the period necessary to simulate a " draw " made by a smoker by means of a cam attached to the periphery of a 3 inch pulley wheel, D. The glass rod was held in the groove of the pulley by means of a stretched rubber band. This wheel was driven by a gear train from a small electric motor, the speed of which was variable by adjustment of a " Variac " auto-transformer connected to the alternating current mains. Other cams could be attached to the wheel and thus the length and frequency of the " draws " were adjusted.

In the U-tubes suitable solvents were placed so that the smoke was made to bubble through them and the condensable material separated from the gas. A packing of 10 cm. of pyrex glass wool in the second U-tube served to remove the last traces of condensable matter. Both U-tubes could be surrounded by beakers full of solid carbon dioxide to assist in the condensation, but it was found that strong cooling was not always necessary to effect complete removal of the disperse phase. An examination of the habits of 150 smokers showed that, with the popular brands of cigarette sold in this country and weighing about 1·1 g. each, the average time of draw is about 2 seconds and the average time of smoking such a cigarette to a short butt (about 1·5 cm. long) is 12 minutes. These conditions are attained by our apparatus when adjusted to give a 2 second draw every 45 seconds with a negative pressure of 25 cm. water. Each cigarette used about 250 ml. air.

Smoke obtained in this way is an aerosol with a viscous liquid as the disperse phase and a gas, consisting of a mixture of unburnt air, carbon dioxide, carbon monoxide, water vapour and traces of other gases as the dispersing medium. The average amount of condensable material from 100 cigarettes weighs about 4 g. We have concerned ourselves only with the condensable disperse phase which in bulk, after the evaporation of the solvent (acetone, chloroform or cyclohexane) used to trap it, is a dark brown viscous fluid.

*Preparation of Apparatus and Materials.*

The solvents (cyclohexane, acetone, benzene and chloroform of reagent grade) were distilled in an all-glass apparatus, rejecting the first and last tenths. This product was then distilled through a Dufton column and collected over a range of $\frac{1}{2}^\circ$ C. leaving one-tenth as residue in the flask. The residues were not fluorescent in ultra-violet light.

All the glass apparatus employed was cleaned by immersing overnight in chromic-sulphuric acid mixture, washing and drying. A blank experiment was performed on the smoking apparatus and solvent by aspiration of 40 litres of air through the absorption tubes containing 5 ml. acetone in each U-tube. This was drained out and the absorption tube washed out with a further 5 ml. acetone. The solution was distilled to dryness in a water bath and the residue redissolved in cyclohexane. The absorption spectrum of this solution was plotted and the presence of approximately 0·02 $\mu$g. pyrene (the most easily determined hydrocarbon in town air) established. This proportion of pyrene is negligible in comparison with the quantities found in the smoking experiments described later.

*Preparation and Use of Chromatographic Columns.*

The columns were prepared as described by Wedgwood and Cooper (1953) in simple glass tubes of diameter about 1 cm. with a short compressed plug of glass wool at the lower end. The alumina was poured into the tube as a thick slurry in the solvent and the tube gently tapped to ensure uniform settling.

Solutions were introduced into the column in as concentrated form as was convenient (not more than 5 ml. in volume) and the separation effected by elution with solvent. Fractions, collected by gravity, were of about 3 ml. in volume, and were examined in the S.P. 500 ultra-violet spectrophotometer. During chromatography the eluates were examined at a few definite wavelengths only and hydrocarbons were identified by specific absorption peaks and the order in which they appeared in the eluates. In quantitative work the eluates containing one compound were combined and re-examined (sometimes after additional chromatography). Fuller details of the method are given in the paper cited above.

*Examination of Smoke.*

It was found that the product of combustion of 50 cigarettes gave a convenient amount of material for further treatment. The dark brown solution was drained out of the apparatus and the latter washed through with more solvent into a flask. The solvent was then removed by distillation, the residue boiled twice with cyclohexane (5 to 10 ml.) and the solutions decanted off after cooling. The united cyclohexane solutions were then shaken with three quantities of 2N sulphuric acid and then with three quantities of 2N sodium hydroxide. The solution thus freed from basic and acidic substances was reduced to about 5 ml. by distillation and chromatographed on activated alumina columns. The eluates from the column were examined by the method of Wedgwood and Cooper (1953) namely by searching for absorption peaks at wavelengths known to be specific for the various polycyclic hydrocarbons. Usually the initial chromatography only revealed the hydrocarbon peaks as inflexions against considerable background absorption and the combined filtrates suspected of containing the compounds had to be passed through fresh columns in order to reveal their presence more satisfactorily. Anthracene was recognised by its peak at 376 m$\mu$. and it was always followed closely by the appearance of a peak at 355 m$\mu$, revealing pyrene.

The compounds were determined by making the cyclohexane solutions up to known volumes and then measuring the height of the peaks by the base line technique. For this purpose two convenient points on either side of a given peak were used to construct a base line. Calibration of the peak heights above the base line was effected by using standard curves of the authentic hydrocarbons prepared from solutions of known strength.

The background absorption interfered considerably in these experiments and various attempts were made to reduce its effect. One of the more successful methods was to evaporate off the cyclohexane and to boil the residue for an hour or so with 2N sodium hydroxide, the solution being re-extracted into cyclohexane. It is thought that much of the interference is due to esters, since some members of this class of compound have been shown to interfere in this way. It was found that the reduction in interference was only effected when treatment with sodium hydroxide was carried out after the hydrocarbons had been separated initially by chromatography. When hydrolysis was effected on the cyclohexane extract of the whole smoke, the background absorption was even greater than before, indicating that some hydrolysis products of the smoke constituents were entering the hydrocarbon sections of the column.

The presence of pyrene and anthracene indicated in these experients was confirmed by their chromatographic behaviour relative to added methyl ethers. Relatively few compounds, absorbing light in the region studied, are associated with the polycyclic hydrocarbons on alumina columns. They are the monomethyl ethers of the hydrocarbons and certain esters. Most other organic compounds with light absorption in this part of the spectrum are much more firmly held on alumina.

Mixtures of known hydrocarbons with suitable methyl ethers in cyclohexane solution give an order of separation on the column, that serves as a standard sequence in an analysis. For example, anisole, anthracene, 1-methoxynaphthalene, pyrene, 2-methoxynapthalene and 3 : 4-benzpyrene separate in that order. The same order of elution was given with the cyclohexane solution of hydrocarbons from cigarette smoke and the hydrocarbons, anthracene and pyrene were again recognised by their characteristic peaks and determined by their peak heights. 3 : 4-Benzyprene was not detected.

Fig. 2 shows portions of the ultra-violet absorption curves of the hydrocarbons and the calibrating ethers in their chromatographic sequence. The peaks used for recognition of the various compounds are labelled with their wavelengths. It will be noted that a sequence of peaks of added calibration materials interspersed with the recognisable peaks of substances originally present, gives an infallible method of identification and determination. The results of a number of analyses utilising in all 250 cigarettes of one popular brand showed quantities of anthracene and pyrene equal to 10·2 and 9·0 $\mu$g. respectively per 100 cigarettes. Although 3 : 4-benzpyrene has not yet been detected we know that there are compounds exhibiting fluorescence in ultra-violet light present in eluates where this hydrocarbon would be expected, and we hope in due course to obtain enough material to identify them. The fact that compounds similar to benzpyrene have been found suggests that the carcinogen might also be present, possibly in quantities too small to be identified with the present scale of working, or so masked by background absorption that it has escaped detection. A considerable amount of coloured material separates on the column above anthracene and pyrene, and it is hoped that this will be resolved into its constituents in due course.

### DISCUSSION.

Previous references to the presence of hydrocarbons in tobacco smoke are few. Wenusch (1937*a*, 1937*b*) and Wenusch and Schöller (1938) refer to " higher " hydrocarbons, but do not specify any definite compound. Wenusch (1934, 1935) also refers to a solid substance melting at 70–72° C. and to paraffins which he states are present in the original tobacco and are vaporised unchanged during smoking.

Acetylene has been determined in tobacco smoke by weighing it as cuprous acetylide (Fishel and Haskins, 1949). Its pyrolysis may give a ready explanation

FIG. 2.—Ultra-violet absorption spectra.

of the presence of the polycyclic hydrocarbons that we have detected, and this explanation is supported by temperature measurements recorded in a later paragraph.

Azulene was obtained by Ikeda (1947) from a green oil obtained by cooling tobacco smoke, distilling the product and subjecting the distillate to chromatography.

It is of interest to consider how polycyclic hydrocarbons such as anthracene and pyrene could be formed in smoke. The presence of various unsaturated compounds has been established ; Berthelot (1886) showed that acetylene formed tarry material upon heating, and isolated benzene and a number of polycyclic compounds from it. Kennaway (1924, 1925) has shown that strong heating of acetylene and other unsaturated materials produced carcinogenic tars at temperatures of 700° C. and above.

We have, therefore, made a study of the temperatures attained in the hot end of a burning cigarette. The use of a disappearing filament pryometer gave only approximate measure of the temperatures because the relatively small glowing end did not fill the field of the instrument. From the brightness of the glowing end during suction it was estimated that a temperature of about 900° C. was attained at the hottest part of the periphery.

More satisfactory measurements were made by using a calibrated copper-nichrome thermocouple which was threaded through the cigarette from one side to the other. After sealing the holes by means of small pieces of paper, the cigarette was smoked in the normal way and the temperature recorded from time to time. The temperature rose rapidly when the burning end advanced towards the couple and, when the glowing material was in contact with the couple, a temperature of about 650° C. was recorded. This remained almost constant while the combustion was continued round the couple and rose about 50° C. each time air was being drawn in. It thus appears that the highest temperatures during suction are confined to the surface and that the main body of the hot end is always at a temperature between 650 and 700° C. Similar temperatures were recorded when a cigarette with a thermocouple inserted was smoked in the mouth. These temperatures are sufficiently high for pyrolysis of simple compounds to polycyclic hydrocarbons. The temperatures recorded by us agree remarkably closely with those obtained independently by Wynder, Graham and Croninger (1953). Our investigations upon the composition of tobacco smoke are being continued.

The authors wish to acknowledge the helpful and kindly criticism given by Professor Sir E. L. Kennaway, F.R.S., the gift of various polycyclic hydrocarbons by Professor J. W. Cook, F.R.S., and a grant from Imperial Chemical Industries Ltd., used to purchase some of the materials and consumable apparatus used in the investigation.

### SUMMARY.

1. Tobacco smoke has been obtained by smoking cigarettes in an automatic apparatus designed to simulate normal human smoking.

2. The whole of the condensable products have been extracted and the neutral extract in cyclohexane has been examined by chromatography followed by absorption spectrophotometry. The extract from 100 cigarettes showed absorption peaks characteristic of anthracene and pyrene and of heights corresponding to 10·2 and 9·0 $\mu$g. respectively.

3. The temperatures attained in the burning end of a cigarette have been measured and were found to fluctuate between 650 and 700° C. These temperatures are sufficiently high to cause pyrolysis of simpler compounds to polycyclic hydrocarbons.

### REFERENCES.

BERTHELOT, M.—(1866) *C. R. Acad. Sci. Paris*, **53**, 481.

BRADFORD, J. A., HARLAN, W. R., AND HANMER, H. R.—(1936) *Industr. Engng. Chem.* **28**, 836. (Contains 10 references to similar work.).

COOPER, R. L.—(1951) M.Sc. Thesis, University of London.—(1953) *Chem. & Ind.* (*Rev.*), 516.
*Idem* AND LINDSEY, A. J.—(1953) *Ibid.*, 1205.
DAFF, M. E., AND KENNAWAY, E. L.—(1950) *Brit. J. Cancer*, **4**, 173.
DOLL, R., AND HILL, A. B.—(1950) *Brit. med. J.*, i. 739.
FISHEL, J. B., AND HASKINS, J. F.—(1949) *Industr. Engng. Chem.*, **41**, 1374.
GROSS, C. R., AND NELSON, O. A.—(1934) *Amer. J. publ. Hlth.*, **24**, 36.
IKEDA, S.—(1947) *Sci. Pap. Inst. phys. chem. Res. Tokyo*, **42**, 8.
KENNAWAY, E. L.—(1924) *J. Path. Bact.*, **27**, 233.—(1925) *Brit. med. J.*, i, 1.
McNALLY, W. D.—(1932) *Amer. J. Cancer*, **16**, 1502.
MILLS, C. A., AND PORTER, M. M.—(1950) *Cancer Res.*, **10**, 539.
RIGDEN, R. H., AND KIRSCHOFF, H.—(1952) *Tex. Rep. Biol. Med.*, **10**, 76.
SADOWSKY, D. A., GILLIAM, A. G., AND CORNFIELD, J.—(1953) *J. nat. Cancer Inst*, **13**, 1237.
SCHREK, R., BAKER, L. A., BALLARD, G. P., AND DOLGOFF, S.—(1950) *Cancer Res.*, **10**, 49.
THOMAS, M. D., AND COLLIER, T. R.—(1945) *J. industr. Hyg.*, **27**, 201.
WEDGWOOD, P., AND COOPER, R. L.—(1951) *Chem. Ind.* (*Rev.*), 1066.—(1953) *Analyst*, **78**, 170.
WENUSCH, A.—(1934) *Biochem. Z.*, **273**, 178.—(1935) *Z. Untersuch. Lebensmitt.*, **69**, 81. (1937*a*) *Ibid.*, **73**, 176.—(1937*b*) *Pharm. Zentralh.*, **78**, 238.
*Idem* AND SCHÖLLER, R.—(1938) *Z. Untersuch. Lebensmitt.*, **75**, 345.
WYNDER, E. L., GRAHAM, E. A., AND CRONINGER, A. B.—(1953) *Cancer Res.*, **13**, 855.